# Supplementary material for: Content-rich biological network constructed by mining PubMed abstracts
Source: BMC Bioinformatics. 2004 Oct 8;5:147. doi: 10.1186/1471-2105-5-147 (PMC528731; doi:10.1186/1471-2105-5-147)
Supplement: Additional File 5 — The original Chilibot query results of the term "long-term potentiation (LTP)" and 22 other terms, limiting the latest references analyzed to the years 1990, 1995, 2000, and 2004. [file 1471-2105-5-147-S5.bz2 › chilibotAdditionalFile5/ltp2004/html/LTP.html]

 


**LTP** (Input: LTP ) 

---


**Google Searches:** Entire Web  | EDU domain only  | PDF files only

.

**External Links:** OMIM |
LocusLink
| Swissprot
| GeneCards

  
**Maps of LTP**

Simple
Complete
graph in
radiant
tree
square
layout.

**New Hypothesis !**

**Synonyms** 

- ltp   [PubMed]

- long term potentiation   [PubMed]

**Synopsis**

- CONCLUSIONS Our results are consistent with models that propose a role for SLP in learning, as mice with decreased PPF or PTP, in the absence of known **LTP** deficits, show profound learning impairments.  Curr Biol, 1996 
  [22]

- Thus, alterations in phospholipid metabolism may play a significant role in the **LTP** deficits observed in FAE offspring.  Alcohol Clin Exp Res, 1997 
  [21]

- These results suggest that glutamate can activate CaM kinase II through NMDA receptors in the induction of **LTP** and in turn stimulates the phosphorylation of target proteins such as MAP2 and synapsin I.  Nippon Yakurigaku Zasshi, 1993 
  [20]

- These results suggest that the increased level of synapsin I mRNA is related to persistent enhancement of synaptic activity within the neural networks in which dentate granule cells participate in **LTP**.  Brain Res, 1998 
  [20]

- In addition, a role for C kinase in long term potentiation  [**LTP**]  has been proposed.  Synapse, 1992 
  [19]

- Such animals have previously been shown to exhibit deficits in NE sensitivity as well as significant impairment in their ability to exhibit **LTP**.  Proc Natl Acad Sci U S A, 1991 
  [19]

- We found that hippocampal long term potentiation  [**LTP**]  was impaired in trkB mice,  Neuron, 2002 
  [18]

- theearly phase of **LTP** is unaffected.  Proc Natl Acad Sci U S A, 2002 
  [17]

- Second, synergy of IP accumulation in correlation with synergy of neurotransmitter release elicited by mAChR activation and membrane depolarization, suggests a possible role for phospholipase C PLC in the bifurcating control of neurotransmitter release and for the involvement of PLC and voltage sensitive channels in mediation of long term potentiation  [**LTP**]  **LTP** .  Neurosci Lett, 1990 
  [16]

- As Group I mGluRs are localized to the perisynaptic region in juxtaposition to NMDARs at glutamatergic synapses, mGluR mediated potentiation of NMDAR activity may play a role in synaptic transmission and plasticity including **LTP**.  Neuropharmacology, 2001 
  [16]

- Glutamate,the most abundant excitatory neurotransmitter in the mammalian CNS, plays a central role in many neuronal functions, such as long term potentiation  [**LTP**] , which is necessary for learning and memory formation.  Prog Brain Res, 2002 
  [16]

- This effect, presumably mediated by effects on calmodulin, is a previously unreported action of cocaine, and suggests that cocaine at high dose levels might disrupt types of learning that are mediated by an **LTP** like mechanism.  Brain Res, 1993 
  [16]

- However, when we examined the presynaptic structure of the hippocampal CA3 field in detail, we found that the sizes of mossy fiber giant terminals were significantly smaller, the number of synaptic vesicles became reduced, and the presynaptic structures altered, although the mossy fiber long term potentiation  [**LTP**]  remained intact.  J Cell Biol, 1995 
  [15]

- These results provide new information about the mechanisms that underlie **LTP** induction and expression and provide evidence for persistent and or calcium independent protein kinase activity involvement in **LTP**.  Learn MemLearn Mem, 1995 
  [11]

- Our results indicate that **LTP** expression is accompanied by persistent changes in presynaptic phosphorylation, and specifically that presynaptic CaM kinase II activity and synapsin I phosphorylation may be involved in **LTP** expression.  Proc Natl Acad Sci U S A, 1996 
  [11]
